# Identification of a novel and high affinity MIF inhibitor *via* structure-based pharmacophore modelling, molecular docking, molecular dynamics simulations, and biological evaluation

**DOI:** 10.1080/14756366.2025.2501378

**Published:** 2025-06-15

**Authors:** Shang Zhu, Shudan Yang, Yao Chen, Miao-Miao Niu, Jun Wang, Jindong Li, Xuehua Pu

**Affiliations:** a Department of Critical Care Medicine, The Affiliated Taizhou People’s Hospital of Nanjing Medical University, Taizhou, China; b Department of Pharmaceutical Analysis, China Pharmaceutical University, Nanjing, China; c Department of Infection Control and Emergency Department, The Affiliated Taizhou People’s Hospital of Nanjing Medical University, Taizhou, China; d Institute of Clinical Medicine, The Affiliated Taizhou People’s Hospital of Nanjing Medical University, Taizhou, China

**Keywords:** MIF, structure-based pharmacophore modelling, molecular docking, MD simulations, biological evaluation

## Abstract

Macrophage migration inhibitory factor (MIF) plays a crucial role in disrupting immune homeostasis and was overexpressed in immune cells. The inhibitors of MIF inhibit the release of inflammatory factors to treat sepsis. Herein, a series of compounds (termed as Hits 1–6) were discovered based on pharmacophore modelling, molecular docking, and interaction analysis. The biaryltriazole inhibitor 3a was used as the positive control. MST and ITC experiments showed that compared to 3a, Hit-1 possessed the highest affinity with MIF. MD simulations exhibited that Hit-1 stably bound to the active pocket of MIF. Pull down experiment showed that Hit-1 could interfere with the binding of MIF to CD74. Furthermore, RT-qPCR demonstrated that Hit-1 suppressed the release of pro-inflammatory cytokines in macrophages including TNF-α, IL-6, and IL-1β. These data demonstrate that Hit-1 may be a promising and high-affinity candidate compound treating sepsis.

## Introduction

Sepsis, caused by the dysregulation of the immune response to infection, has become a major challenge to the healthcare system^1^. It is a heterogeneous disease caused by multiple factors and possesses the high morbidity and mortality rates^2^. It is arduous to diagnose and treat sepsis among patients who have gone through surgical procedures^3^. The pathogenesis of sepsis is complex and the sepsis progresses rapidly^4^^,^^5^. Sepsis can further progress to septic shock, leading to multiple organ dysfunction and even death in severe cases^6^. Immunosuppression, lactic acidemia, and lung injury are induced by the sepsis, and are the major causes of high mortality^7^^,^^8^. Sepsis is treated clinically with antibiotics, fluid resuscitation, glucocorticoids etc^9^^,^^10^. The antimicrobial treatment suffers from drug resistance and antibiotic misuse^11^. Currently, the prognosis of the treatment methods for sepsis is rather poor, with high costs and frequent recurrence^12^^,^^13^. Therefore, the new strategies for the treating sepsis are urgently needed.

MIF, which belongs to the MIF family, consists of a ring trimer protein composed of 342 residues^14^. MIF has the direct pro-inflammatory role in inflammatory diseases, such as sepsis^15^. It has been demonstrated to be overexpressed in both immune and non-immune cell types, with macrophages, endothelial cells, and T cells being among the typical representatives^16^. When pathogenic microorganisms with lipopolysaccharide (LPS) as a key virulence factor infect host cells, immune cells are activated to release MIF^4^. MIF binds to receptors CD74/44 and CXCR2, 4, 7 to exert its effects in an autocrine and paracrine manner^17^^,^^18^. Therefore, it activates ERK1/2, AMPK, and AKT signalling pathways to mediate the effects of other pro-inflammatory factors, including interleukins, interferons, and NF-kB^19–21^. Excessive releases of pro-inflammatory factors lead to disruption of immune homeostasis resulting in sepsis^21^. Cumulative clinical data suggest that MIF is an attractive target for multiple inflammatory and autoimmune diseases^22^. The inhibitors of MIF for sepsis have attracted the attention of a number of researchers.

Currently, inhibitors of MIF are generally classified into three categories^23^: (1) Inhibitors that covalently modify the N-terminal Pro1 residue; (2) Inhibitors that bind to the catalytically active site; (3) Inhibitors that disrupt the structure of MIF trimers; A biaryltriazole inhibitor 3a is potent MIF inhibitor. The X-ray crystal structure of the complex with the MIF tautomerase active site and compound 3a has been revealed, and it serves as the positive control drug^24^. The IPG1094 is the first inhibitor of MIF to be used in clinical trials worldwide, approved by the FDA and the State Drug Administration in China. It has shown good therapeutic effects on a variety of immune disorders^22^. NAPQI, PMSF, 4-IPP, ISO-1, OXMI-11 are inhibitors of MIF^23^^,^^25^. However, the inhibitors possess poor efficacy, high liver and kidney toxicity^17^. Currently, there are no dedicated therapeutic agents for sepsis in the clinic^26^. Therefore, the development of a novel, high-affinity small molecule compound targeting MIF is urgent and important matter.

Computer-based virtual screening plays a vital role in screening lead compounds to treat diseases^27^^,^^28^. Pharmacophore modelling rests upon optimal interrelationships between ligands and proteins^29^. Pharmacophore modelling and docking screening methods fully consider protein flexibility, solvation effects, ligand conformation, etc^30^. In previous studies, we found some target inhibitors by virtual screening based on pharmacophore modeling^31–36^. In this study, we successfully identified of a novel and high affinity MIF inhibitor (Hit-1) by structure-based pharmacophore modelling, molecular docking, MD simulations, and biological evaluation.

## Results

### Pharmacophore model of MIF inhibitors

**Figure 1.**
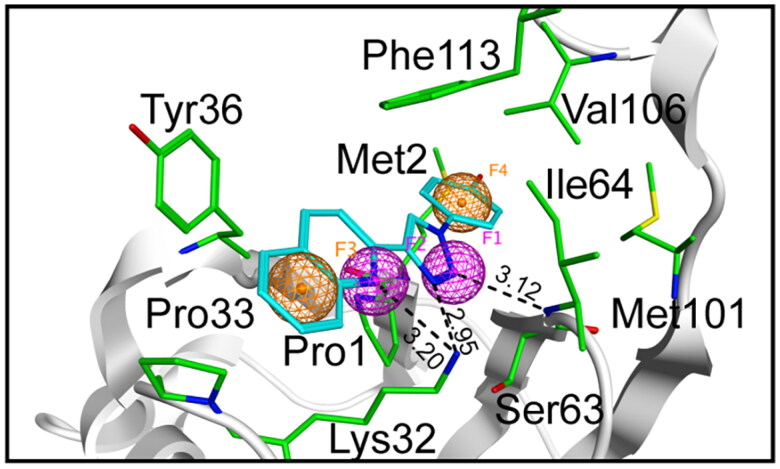
The pharmacophore model of the active pocket of MIF. The amino acids were depicted as sticks with atoms coloured carbon-green, oxygen-red, nitrogen-blue, and sulfur-yellow. The compound 3a was depicted in cyan. The pharmacophore model was composed of F1 (hydrogen bond donor feature), F2 (hydrogen bond donor feature), F3 (aromatic feature), and F4 (aromatic feature). The backbone of the MIF was indicated by the gray. The hydrogen bonds were represented by the black dotted line. The hydrogen bond distance was labelled in the figure. The unit of hydrogen bond distance was angstrom (Å).

The crystal structure of MIF in complex with the compound 3a was imported into the molecular operating environment (MOE, Chemical Computing Group Inc, Montreal, Quebec, Canada) software. The Ligand Interaction tool was used to analyse the interaction relationship between the compound 3a and MIF. In the active pocket of MIF, Tyr36, and Phe113, as the key amino acid residues, participated in π-π interactions. Concurrently, Lys32 and Ile64 residues interacted with compound 3a through hydrogen bonding. Based on the interaction relationship, the pharmacophore model was constructed using the pharmacophore editor. As shown in Figure 1, pharmacophore features F1 (Acc) and F2 (Acc) were established in accordance with hydrogen bonding interactions. Likewise, pharmacophore features F3 (Aro) and F4 (Aro) were structured on the basis of π-π interactions. These pharmacophore features jointly constituted the pharmacophore model of the MIF inhibitors. The constructed pharmacophore model was used to screen out the active compounds.

### Structure-based virtual screening of compounds

**Figure 2.**
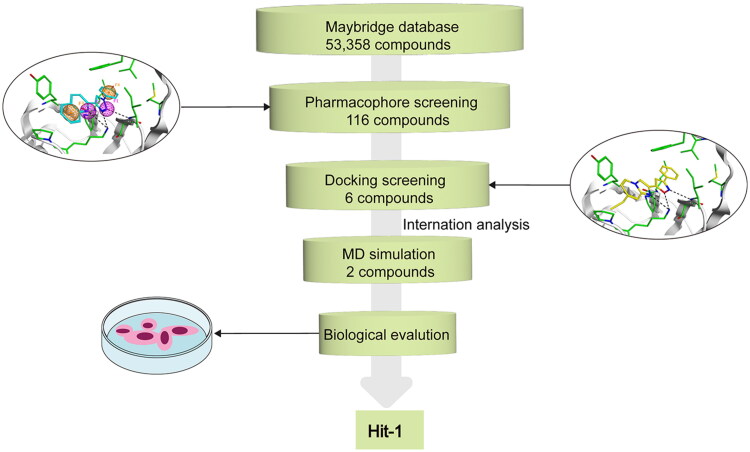
A flowchart of the integrated screening process of MIF inhibitors.

**Figure 3.**
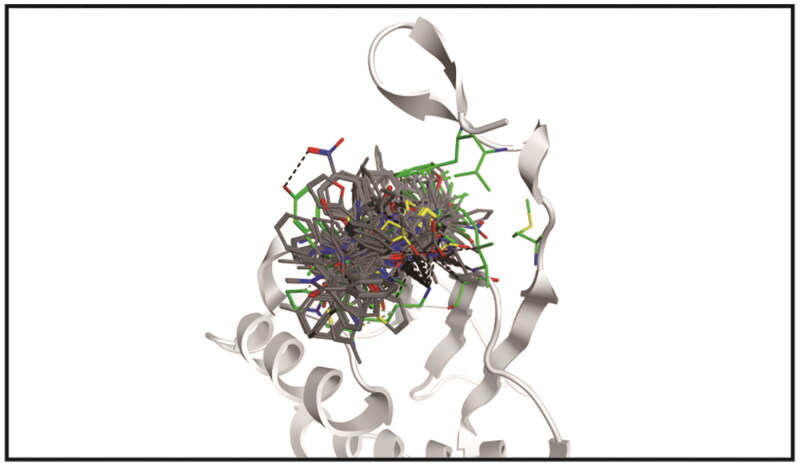
The diagram of 116 compounds docked into the active pocket of the MIF structural domain.

**Figure 4.**
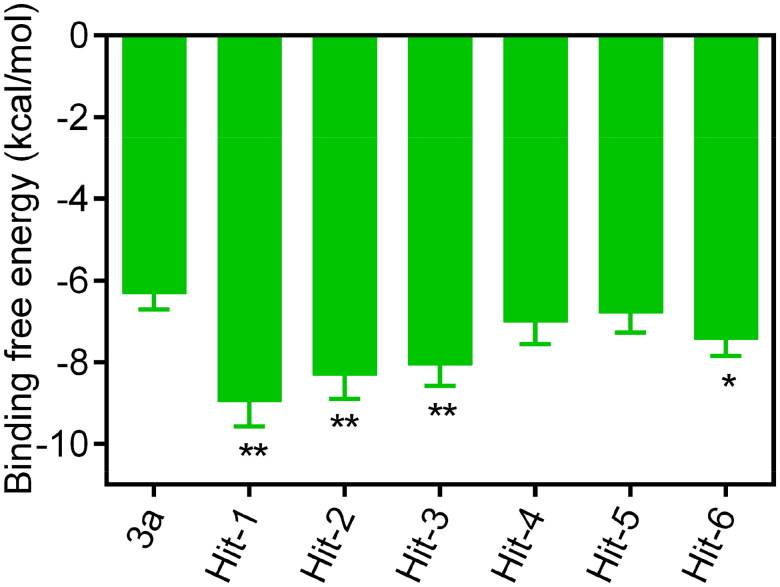
The binding free energies (kcal/mol) of the compounds 3a and Hits 1–6. Significance: *p < 0.05, **p < 0.01.

**Figure 5.**
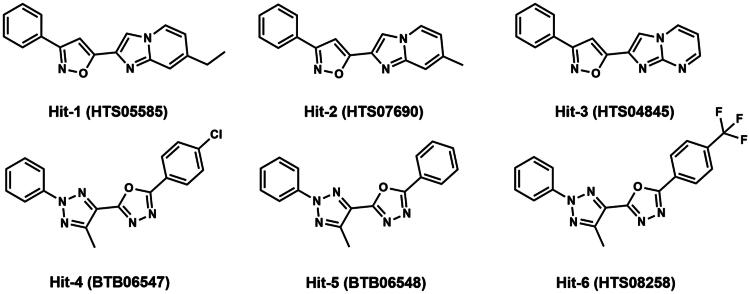
The chemical structures of Hits 1–6. The catalogue numbers of the compounds in the Maybridge database were indicated.

A flowchart of the multistep screening was presented in Figure 2. The Maybridge database containing 53 358 compounds was selected for virtual screening. The energy minimisation algorithm was used to transform compounds from 2D to 3D structures. Based on the constructed pharmacophore model, 116 compounds were screened from the database. Then, they were docked to the active pocket of the MIF structural domain, and Figure 3 showed the docking diagram. Moreover, the structures were observable in Figures S1.1–1.3. Based on the docking scores, the top six compounds were selected and termed as Hits 1–6. As shown in Table S1, the docking scores of Hits 1–6 were obtained using the GBVI/WSA dG scoring function. In parallel, Figure 4 represented the binding free energies (measured in kcal/mol) of compound 3a and Hits 1–6, providing a visual reference for the interactions. Figure 5 demonstrated the chemical structures of Hits 1–6. Next, we proceeded with an in-depth interaction analysis of the docking modalities between Hits 1–6 and MIF. Then, we carried out microscale thermophoresis (MST) experiments on Hits 1–6 and compound 3a to measure *K_d_* values and assess their affinity to MIF. The isothermal titration calorimetry (ITC) experiments confirmed this result. Moreover, Hit-1 and Hit-2 (excellent compounds) were selected for 50 ns molecular dynamics (MD) simulations to assess the stability binding to MIF. The pull down and reverse transcription quantitative polymerase chain reaction (RT-qPCR) experiments were carried out for the biological evaluation of Hit-1. Eventually, we successfully identified of a novel and high affinity MIF inhibitor (Hit-1) by structure-based pharmacophore modelling, molecular docking, MD simulations, and biological evaluation.

### Interaction analysis

**Figure 6.**
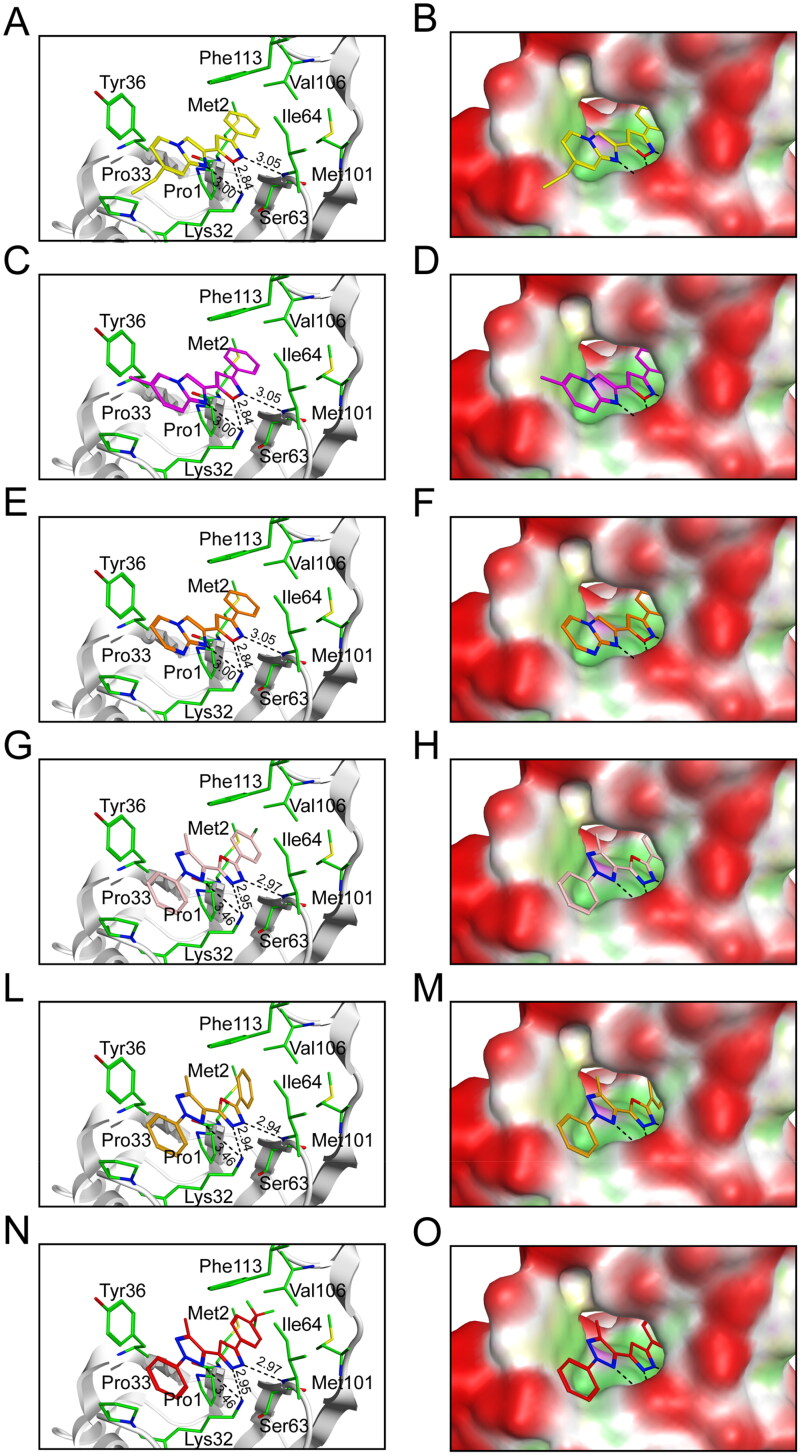
The binding modes and binding surfaces of Hits 1–6 at active sites of MIF. (A,B) Prediction for Hit-1 in MIF. Hit-1 was shown in yellow. (C,D) Prediction for Hit-2 in MIF. Hit-2 was colour-coded by purple. (E,F) Prediction for Hit-3 in MIF. Hit-3 was represented in orange. (G,H) Prediction for Hit-4 in MIF. Hit-4 was shown in pink. (L,M) Prediction for Hit-5 in MIF. Hit-5 was colour-coded by brown. (N,O) Prediction for Hit-6 in MIF. Hit-6 was represented in red. The hydrogen bond distance was labelled in the figure.

The docking models of Hits 1–6 with the active pocket of the MIF were meticulously analysed using the Ligand Interaction tool. The Hits 1–6 exhibited a high degree of complementarity with the active pocket of MIF. The stronger the interaction between the Hits 1–6 and MIF, the more stable their binding modes. As illustrated in Figures 6(A–F), the imines present in the five elemental rings of Hits 1–3 functioned as hydrogen donors, thereby forming hydrogen bonding interactions with the key residues, Lys32 and Ile64, located within the active pocket of MIF. The oxygen atoms harboured within the five elemental rings of Hits 1–3 served as hydrogen acceptors to form hydrogen bonding interactions with residue Lys32. Figures 6(G–O) depicted that nitrogen atoms in the five membered rings formed three hydrogen bonds with residues Lys32 and Ile64 in MIF-Hits 4–6 complexes, respectively. The shorter the hydrogen bond distance, the stronger the hydrogen bonding interaction. The residues Tyr36 and Phe113 engaged in π-π interactions with Hits 1–6. The residues Pro33, Pro1, Met2, Ser63, Met101, and Val106 formed the hydrophobic pocket in the MIF-Hits 1–6 complexes, respectively. The structure-based interaction analysis provided the better understanding of the stability of Hits 1–6 binding to MIF. The stronger the interaction, the higher the binding affinity.

### Affinity determination

The MST experiment was used to measure the *K_d_* values of the compound 3a and Hits 1–6 to evaluate the binding affinity to MIF. The lesser the *K_d_* value, the greater the affinity that the compound exhibited for the MIF. The compound 3a, as a positive control, exhibited a *K_d_* value of 0.87 ± 0.09 μM (Table 1). Compared to the compound 3a, the *K_d_* values of the Hits 1–6 were all lower than the *K_d_* value of compound 3a. Among all the hit compounds, Hit-1 possessed the lowest *K_d_* value of 0.29 ± 0.01 μM. Therefore, Hit-1 manifested the strongest affinity towards MIF. In conclusion, Hits 1–6 stably bound to MIF with the high affinity and Hit-1 was the optimal compound. Next, we selected Hit-1 and compound 3a for the ITC experiment to verify the reliability of the results. The *K_d_* value of compound 3a was 0.94 ± 0.07 μM and the *K_d_* value of Hit-1 was 0.32 ± 0.01 μM (Table S2). The experimental results revealed that, compared with compound 3a, Hit-1 had a lower *K_d_* value, indicating a stronger affinity for MIF. Thus, the MST and ITC experiments demonstrated that Hit-1 bound firmly to MIF’s active site.

**Table 1.** Analysis of the binding affinity between Hits 1–6, 3a, and MIF by MST assay.

Name	MIF (*K_d_*, µM)
Hit-1	0.29 ± 0.01
Hit-2	0.46 ± 0.03
Hit-3	0.54 ± 0.02
Hit-4	0.75 ± 0.06
Hit-5	0.81 ± 0.04
Hit-6	0.72 ± 0.05
3a	0.87 ± 0.09

### MD simulations

**Figure 7.**
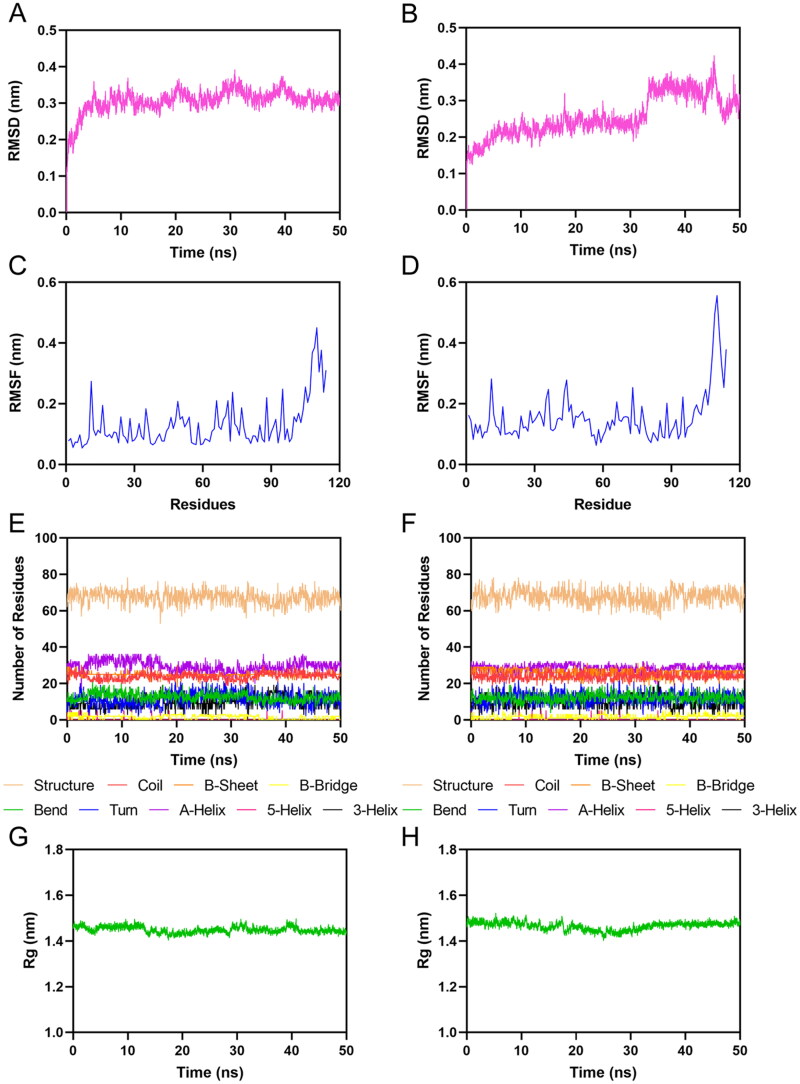
MD simulations of MIF in complex with Hit-1 and Hit-2. (A) The RMSD plot of MIF-Hit-1 complex. (B) The RMSD plot of MIF-Hit-2 complex. (C,D) The RMSF plots of Cα-atoms of residues of MIF in MIF-Hit-1 and MIF-Hit-2 complexes, respectively. The secondary-structure contents of MIF in MIF-Hit-1 (E) and MIF-Hit-2 (F) complexes. The Rg plots of MIF in MIF-Hit-1 (G) and MIF-Hit-2 (H) complexes.

Given the outstanding performance of Hit-1 and Hit-2 in the MIF affinity tests, the 50 ns MD simulations were conducted to assess their binding stability to MIF based on four metrics. The root-mean square deviation (RMSD) plot illustrated the difference between the structure of the complex and the initial structure over time. The MIF-Hit-1 complex system had the average RMSD value of 0.3 nm during the 50 ns MD simulation (Figure 7(A)). The MIF-Hit-2 complex system averaged 0.22 nm within the first 33 ns and then stabilised at 0.34 nm (Figure 7(B)). The smaller the RMSD value, the more stable the complex system during the simulation. The root-mean square fluctuation (RMSF) plot exhibited the flexibility of key residues in the active pocket of the MIF during the simulation. The crucial residues included Pro1, Met2, Lys32, Pro33, Tyr36, Ser63, Ile64, Met101, Val106, and Phe113. The RMSF values of the MIF-Hit-1 complex were all below 0.24 nm (Figure 7(C)). The RMSF values of the MIF-Hit-2 complex were <0.26 nm (Figure 7(D)). The secondary-structure plot represented the stability of the secondary structure of the MIF. The stable lines inhibited stable complex systems. The most MIF residues were composed of structural and A-helical structures (Figures 7(E,F)). The radius of gyration (Rg) plot showed the overall compactness of the protein during the simulation. The stable Rg value indicated that the protein structure maintained overall compactness. The Rg values of MIF were stabilised at 0.145 nm in MIF-Hit-1 complex (Figure 7(G)). The Rg values of MIF were stable at 0.15 nm within the MIF-Hit-2 complex (Figure 7(H)). In summary, compared to Hit-2, Hit-1 bound more stably with the active pocket of MIF.

### Pull down analysis

Taking into account that Hit-1 had the ability to bind stably to MIF, the pull down was implemented for the biological evaluation of Hit-1. Pull down assay with GST-MIF and CD74 showed differential bands, and western blot (WB) was performed to further confirm the accuracy and reliability of the experiment. Results revealed that GST-MIF pull-down of CD74 exhibited a dose-dependent reduction as CD74 concentration increased (Figure S3). MIF directly interacted with CD74, and this interaction was potently suppressed by Hit-1. In conclusion, Hit-1 was capable of blocking the binding between MIF and its receptor CD74.

### RT-qPCR

**Figure 8.**
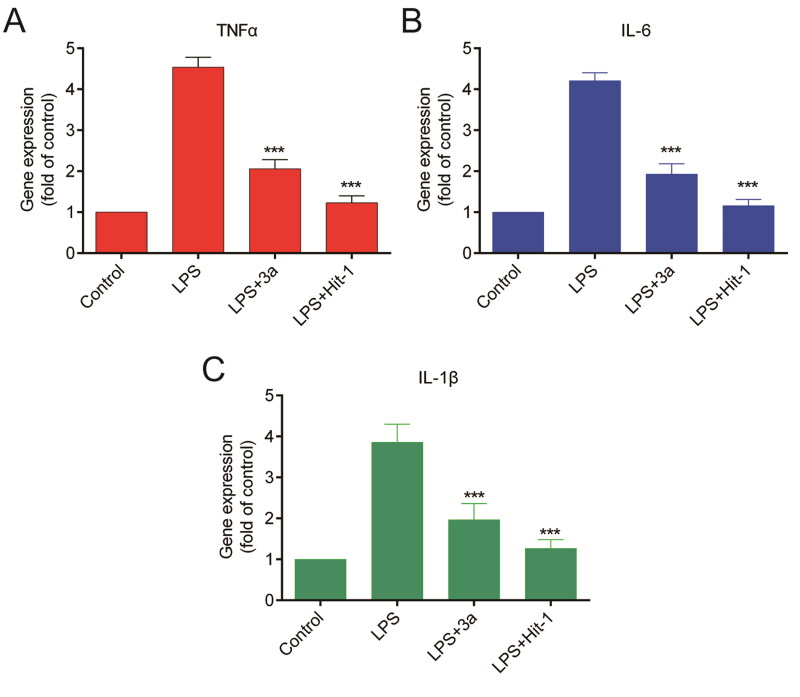
Effects of Hit-1 and compound 3a on LPS-induced proinflammatory genes expression at mRNA levels in RAW 264.7 macrophages. The mRNA expression of TNF-α (A), IL-6 (B), and IL-1β (C) were verified using SYBR green quantitative RT-PCR analysis. Significance: ***p < 0.001.

The inhibitors of MIF (Z-312, ISO-1) could all suppress the release of pro-inflammatory factors induced by lipopolysaccharide (LPS) including tumour necrosis factor -α (TNF-α), interleukin-6 (IL-6), and IL-1β^37,^^38^. The gene expression levels of pro-inflammatory factors in normal cells were represented by the Control group. In the LPS group, cells released a large amount of pro-inflammatory factors under the induction of LPS. As can be seen from Figure 8(A), compound 3a was able to reduce the TNF-α level by 2.2-fold compared with the LPS group. The Hit-1 group significantly reduced the TNF-α level by 3.7-fold compared with the LPS group. Compared with compound 3a, the Hit-1 group exhibited a stronger inhibitory effect on the TNF-α level. It was deducible from Figure 8(B) that the Hit-1 group manifested a markedly stronger inhibitory effect on the IL-6 level. As shown in Figure 8(C), the Hit-1 group demonstrated a distinctly stronger inhibitory effect on the IL-1β. In summary, compared with compound 3a, the Hit-1 group could effectively suppress the release of pro-inflammatory factors (TNF-α, IL-6, and IL-1β) induced by LPS.

## Discussion

Sepsis is a state of persistent systemic inflammatory response involving multiple organ failure^6^. Most cases of sepsis are caused by Gram-negative (G^−^) infections^11^. Clinical studies have shown that patients with sepsis have high levels of MIF in blood^6^. Overexpression of MIF is closely related to the severity of the disease in immune cells^4^. When pathogenic microorganisms infect host cells, immune cells release MIF, which promotes the release of other pro-inflammatory factors^19^. The excessive release of inflammatory factors leads to the disruption of immune homeostasis^20^. Inhibitors of MIF can block the binding of MIF to the CD74 receptor, thereby inhibiting the release of pro-inflammatory factors^25^. Moreover, clinical data indicate that MIF is served as a biomarker for the treatment and diagnosis of sepsis^3^. Currently, most inhibitors of MIF have significant liver and kidney toxicity^17^. More importantly, there are no specific drugs for the treatment of sepsis in clinical practice^26^. Therefore, the discovery of novel high-affinity small molecule compounds targeting MIF has been drawing extensive attention.

The crystal structure of MIF in complex with a biaryltriazole inhibitor 3a (PDB ID: 4WR8) was downloaded from the PDB database. The construction of pharmacophore modelling was founded upon the interactive modalities between MIF and compound 3a. In particular, F1 and F2 functioned as hydrogen bond donor characteristics, with F3 and F4 serving as aromatic traits. We selected the Maybridge database containing 53 348 compounds for virtual screening. The 116 compounds were screened based on pharmacophore modelling. The 116 compounds were docked to the active pocket of the MIF structural domain. Based on the docking scores, the top six compounds were selected for the following experiments. Interaction analysis showed that hydrogen bond formation and hydrophobic pockets were essential for the compounds to bind to MIF. The key residues in the active pocket of the MIF included Pro1, Met2, Lys32, Pro33, Tyr36, Ser63, Ile64, Met101, Val106, and Phe113.

Hits 1–3 featured an isoxazole moiety, whereas Hits 4–6 incorporated a triazole moiety. The hydrogen bonds formed by O-H in Hits 1–3 had a distance of 2.84 Å, whereas the hydrogen bonds formed by N-H in Hits 4–6 exhibited distances of 2.95 or 2.94 Å. The shorter hydrogen bond distances indicated the stronger hydrogen bond interactions. Compared to Hits 4–6, Hits 1–3 possessed the shorter hydrogen bond distance and formed the stronger interaction with the residue Lys32, which was the most critical amino acid residue in the MIF active site. The structural differences among Hits 1–3 lied in the substituents attached to the isoxazole ring: Hit-1: ethyl group; Hit-2: methyl group; Hit-3: hydrogen atom; Among Hits 1–3, Hit-1 displayed the strongest hydrophobicity due to the hydrophobic ethyl group. Hit-1 formed hydrophobic interactions with the MIF active site residues Pro33, Pro1, Met2, Ser63, Met101, and Val106. Hit-1 exhibited the lowest docking score (Hit-1 = −8.96 ± 0.61 kcal/mol). MST and ITC experiments exhibited that compared to compound 3a and Hits 2–6, Hit-1 possessed the highest affinity with MIF. MD simulations exhibited that Hit-1 stably bound to the active pocket of MIF. Moreover, pull down experiment demonstrated that Hit-1 could effectively inhibit the binding between MIF and its receptor CD74. Finally, the RT-qPCR assay demonstrated that compared to compound 3a, Hit-1 inhibited LPS-induced TNF-α, IL-6, and IL-1β release in macrophages. These data demonstrate that Hit-1 may be a promising and high-affinity candidate compound treating sepsis.

## Conclusion

While MIF overactivation is a validated therapeutic target in sepsis, clinical utility of existing inhibitors is hindered by adverse reactions. It is urgent to discovery a new MIF inhibitor to treat sepsis. Using the pharmacophore editor, pharmacophore model of MIF inhibitors was constructed based on the interaction relationship between the biaryltriazole inhibitor 3a and the active site of MIF within MOE. Using the established pharmacophore model, 116 compounds were screened from the Maybridge database (53 348 compounds). These compounds were docked to the active site of MIF, and the top six compounds (named Hits 1–6) were selected based on docking scores. The binding modes analysis were performed for Hits 1–6 interacting with MIF. The affinity measurement experiments revealed that Hit-1 exhibited the strongest binding affinity with the *K_d_* value of 0.29 ± 0.01 μM (MST assay) and the *K_d_* value of Hit-1 is 0.32 ± 0.01 μM (ITC assay). MD simulations demonstrated stable binding of Hit-1 to MIF. Pull down assay showed that Hit-1 was capable of blocking the binding between MIF and its receptor CD74. RT-qPCR experiments further confirmed that Hit-1 suppressed the release of pro-inflammatory cytokines in macrophages including TNF-α, IL-6, and IL-1β. In conclusion, a novel and high affinity MIF inhibitor (Hit-1) was identified *via* structure-based pharmacophore modelling, molecular docking, molecular dynamics simulations, and biological evaluation.

## Materials and methods

### General

All chemicals were purchased from commercial suppliers without additional purification required. A biaryltriazole inhibitor 3a (CAS number: 1663475-08-8) was purchased from MedChemExpress (Monmouth Junction, NJ, USA). Hits 1–6 compounds were obtained from WuXi AppTec (Shanghai, China) with a purity exceeding 98%. The high-performance liquid chromatography (HPLC) spectra data for each compound were provided in the Supplementary Materials (Figures S2.1–2.6). RAW 264.7 murine macrophages were purchased from the American Type Culture Collection (ATCC, TIB-71). 10% foetal bovine serum (FBS), 100 units/mL of penicillin, and 100 µg/mL of streptomycin were added to the Dulbecco’s modified Eagle’s medium (DMEM). The FBS, penicillin, streptomycin, and DMEM were purchased from Gibco (Grand Island, NY, USA). The cells were cultured in the incubator that was humidified with 5% CO_2_ and 95% air at 37 °C. The human recombinant MIF was purchased from Abcam (Cambridge, MA, USA) and catalogue number was ab51096.

### Construction of pharmacophore for MIF inhibitors

The crystal structure of MIF in complex with the biaryltriazole inhibitor 3a (PDB ID: 4WR8), characterised by a resolution of 2.60 Å, was downloaded from the Protein Data Bank (PDB) database. It was introduced into the MOE software. The Ligand Interaction tool was used to analysis the interaction between MIF and 3a. Based on the interaction relationships, the pharmacophore editor was used to edit the pharmacophore features. Pharmacophore features were taken into account including hydrogen bond donors, hydrogen bond acceptors, and aromatic centres. These pharmacophore characteristics then formed the pharmacophore model. Pharmacophore model contained the key information binding to the active pocket of MIF. The constructed pharmacophore model was used to screen a number of active compounds from the database.

### Virtual screening

The Maybridge database, containing 53 358 compounds, was used for virtual screening. By utilising the energy minimisation algorithm, the compounds were converted from their 2D structures to 3D structures, which was conducive to better docking with the active site of the MIF protein. The constructed pharmacophore model was used as initial screening. As a result of the screening based on pharmacophore modelling, the obtained compounds were docked with the active pocket of the MIF protein. We used the Triangle Matcher algorithm and London dG scoring for the first scoring. The Rigid Receptor algorithm and the GBVI/WSA dG scoring were used for the second scoring. Interaction analysis was performed based on the top 6 scoring compounds (termed as Hits 1–6). MST and ITC experiments were performed to measure the binding affinities of Hits 1–6 to MIF. MD experiments were performed to evaluate whether Hit-1 and Hit-2 bind stably to MIF. Pull down and RT-qPCR experiments were conducted to assess the effects of Hit-1 in sepsis. Based on virtual screening, we screened a series of active compounds binding to MIF protein.

### Interaction analysis

The binding modes of each of Hits 1–6 in relation to the active pocket of MIF were separately incorporated into the MOE software. Ligand Interaction tool was utilised for interaction analysis. We focused on the formation of hydrogen bonds, hydrophobic interactions, and π-π interactions in key residues of the active pocket of MIF. The distances of hydrogen bonds formed between Hits 1–6 and the crucial residues of MIF were noted. A shorter hydrogen-bond distance implied a stronger interaction^39^.

### MST assay

The MST assay was used to measure the *K_d_* value, aiming to assess the binding affinity between small molecules and targeted proteins in the field of drug discovery^40^. The compound 3a was used as the positive control. The degree of labelling (DOL) of the MIF protein was calculated. The ideal DOL was in the range of 0.5–1. The Monolith™ RED-NHS second-generation protein labelling kit (NanoTemper Technologies) was used for the fluorescent labelling of MIF protein. We strictly followed the instructions of the kit for the experiment. The PBST (Phosphate-Buffered Saline with Tween) solution containing 0.05% Tween 20 was configured. The ligand was diluted into 16 concentration gradients using the semi-dilution method. The fluorescently labelled protein was added and thoroughly mixed, then incubated for 5 min in the dark. The solution was taken by capillary tube and assayed with the Monolith NT.115 instrument (NanoTemper Technologies). Subsequently, the difference was recorded in fluorescence intensity. The fluorescence intensity was plotted against the concentration of the hit compounds to generate a fluorescence dose curve, following which the *K_d_* value was calculated.

### ITC assay

ITC experiments were performed as described previously^41^. This study employed a VP-ITC titration calorimeter (Microcal, Inc., Northampton, MA, USA). The buffer (pH 7.5) was prepared by combining 20 mM Tris (hydroxymethyl) aminomethane chloride (Tris·HCl), 100 mM NaCl, and 3 mM Dithiothreitol (DTT). A 5 μL aliquot of Hit-1 was added dropwise to the MIF solution using a syringe and then mixed thoroughly. At this moment, the titration temperature was maintained at 25 °C, and the stirring speed was set to 300 rpm. The Hit-1 titration buffer was utilised as a blank control for rectifying the influence that the dilution and buffer effects had on the affinity results. The Origin ITC analysis software was used to fit the corrected isotherm. The titration data were subjected to fitting, and the errors were computed by means of the nonlinear least squares method. In line with the structural data, a 1:1 stoichiometry was hypothesised, and the data were then fitted to a single-site binding model. The *K_d_* value of the binding affinity was obtained from the binding curve.

### MD simulations

The binding modes of Hit-1 and Hit-2 with the active pocket of MIF were separately imported into the MOE software. The QuickPrep tool was used to pre-process the complex system. We initially deleted the ligand to obtain the file of the protein in pdb format. We removed the protein sequence to retrieve the file of the ligand in pdb format. The pdb file of the ligand was submitted to the site for computation (https://www.bio2byte.be/acpype/). The 50 ns MD simulations were carried out under the AMBER99SB-ILDN force field. The ligand file was added to the protein file to get the complex file. The complex was solvated in the cube with the diameter of 1.0 nm. The counteracting ions (sodium and chloride ions) were added to bring the complex system into equilibrium. The 5000-step steepest algorithm was used for energy minimisation. The nvt equilibrium was carried out at the temperature of 300 K. Subsequently, we performed the npt equilibrium (pressure of 1.0 bar). RMSD, RMSF, secondary-structure contents, and Rg were calculated during the 50 ns simulation process. The trajectory was recorded every 10 ps.

### Pull down assay

The pull down experiment was executed as described previously^36^ and in line with defined standard principles. The pre-washed beads with purified GST-MIF were incubated for 2 h at 4 °C. The sample was centrifuged at 10 000 rpm and the purified protein was transferred to a new Eppendorf (EP) tube to remove non-specifically bound background proteins. The 40 nM of biotin-Hit-1 was added to the purified protein for incubation. Then, the pre-washed beads were added to the incubated samples (in the absence or presence of 100 nM and 200 nM of CD74) overnight. The beads were washed five times with PBST. The proteins were denatured at 100 °C for 5 min. The protein bands were isolated and segregated *via* sodium dodecyl sulphate-polyacrylamide gel electrophoresis (SDS-PAGE) technique. The polyvinylidene fluoride (PVDF) membrane was pre-soaked in methanol. The protein bands were transferred onto the PVDF membrane under the condition of 200 mA. The membrane was blocked by incubating it with 3% bovine serum albumin (BSA) in PBS at 37 °C for 2 h. After every incubation period ended, the membrane was washed three times successively with PBST, with each washing step taking 10 min. Subsequently, the membrane was subjected to incubating with the primary antibody on a shaker overnight at 4 °C. The membrane was incubated jointly with the HRP-conjugated secondary antibody at 37 °C for 1 h. The ECL chemiluminescent substance was carefully smeared onto the membrane to ensure even coverage. Thereafter, the light signal was detected by a sophisticated detection system. Employing the Image J software, the intensity of the bands was quantified.

### RT-qPCR

This experiment was carried out in accordance with the relevant literature^37^. This experiment was divided into four groups: a blank control group (Control), a negative control group (LPS), a positive control group (LPS + 3a), and a Hit-1 group (LPS + Hit-1). RAW 264.7 macrophages were subjected to treatment with LPS at a concentration of 200 ng/mL for 6 h. After induction, the cells underwent incubation in combination with 300 nM Hit-1 or compound 3a for 30 min. The Trizol method was used to extract the total ribonucleic acid (RNA) from the cells. The RNA reverse transcription kit (Invitrogen) was used to synthesise complementary deoxyribonucleic acid (cDNA) according to the manufacturer’s instructions. The SYRB Green qPCR kit and the primers were used for amplification. Thereafter, to standardise the data, the expression levels of the target genes were normalised to GAPDH and calculated by using the 2^−ΔΔCT^ method. The expression data were presented in the format of fold-change for clear visualisation.

### Statistics analysis

The data processing and graphing were both done with GraphPad Prism software (version 9.5). The *t*-tests were utilised to ascertain whether there was a significant difference between the two sets of data. The *p*-values lower than 0.05 were deemed to be statistically significant. Each experiment was repeated three times. The data were presented as the mean ± standard deviation (*SD*).

## Supplementary Material

Supplemental_Material_for_review_ Clean.doc

## Data Availability

The data presented in the current study are available from the corresponding author upon reasonable request.
