# Supplementary material for: Identification of a novel and high affinity MIF inhibitor via structure-based pharmacophore modelling, molecular docking, molecular dynamics simulations, and biological evaluation
Source: J Enzyme Inhib Med Chem. 2025 Jun 15;40(1):2501378. doi: 10.1080/14756366.2025.2501378 (PMC12172083; doi:10.1080/14756366.2025.2501378)
Supplement: Supplemental_Material_for_review_ Clean.doc [file IENZ_A_2501378_SM1016.doc]

**Identification of a novel and high affinity MIF inhibitor via structure-based pharmacophore modeling, molecular docking, molecular dynamics simulations and biological evaluation**


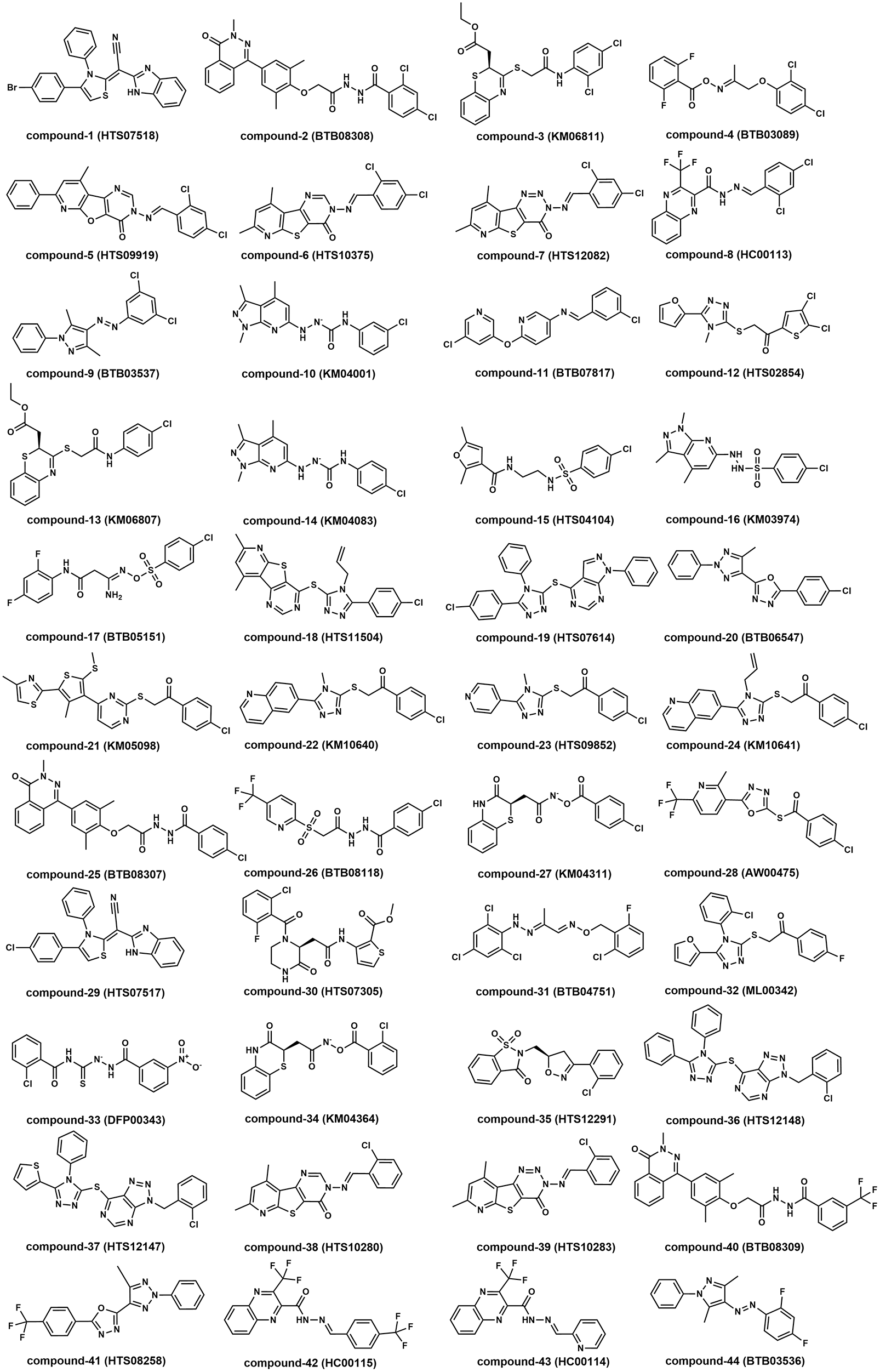


Figure S1.1. The structures of compounds 1 - 44 among the 116 compounds screened out by the pharmacophore model of MIF.


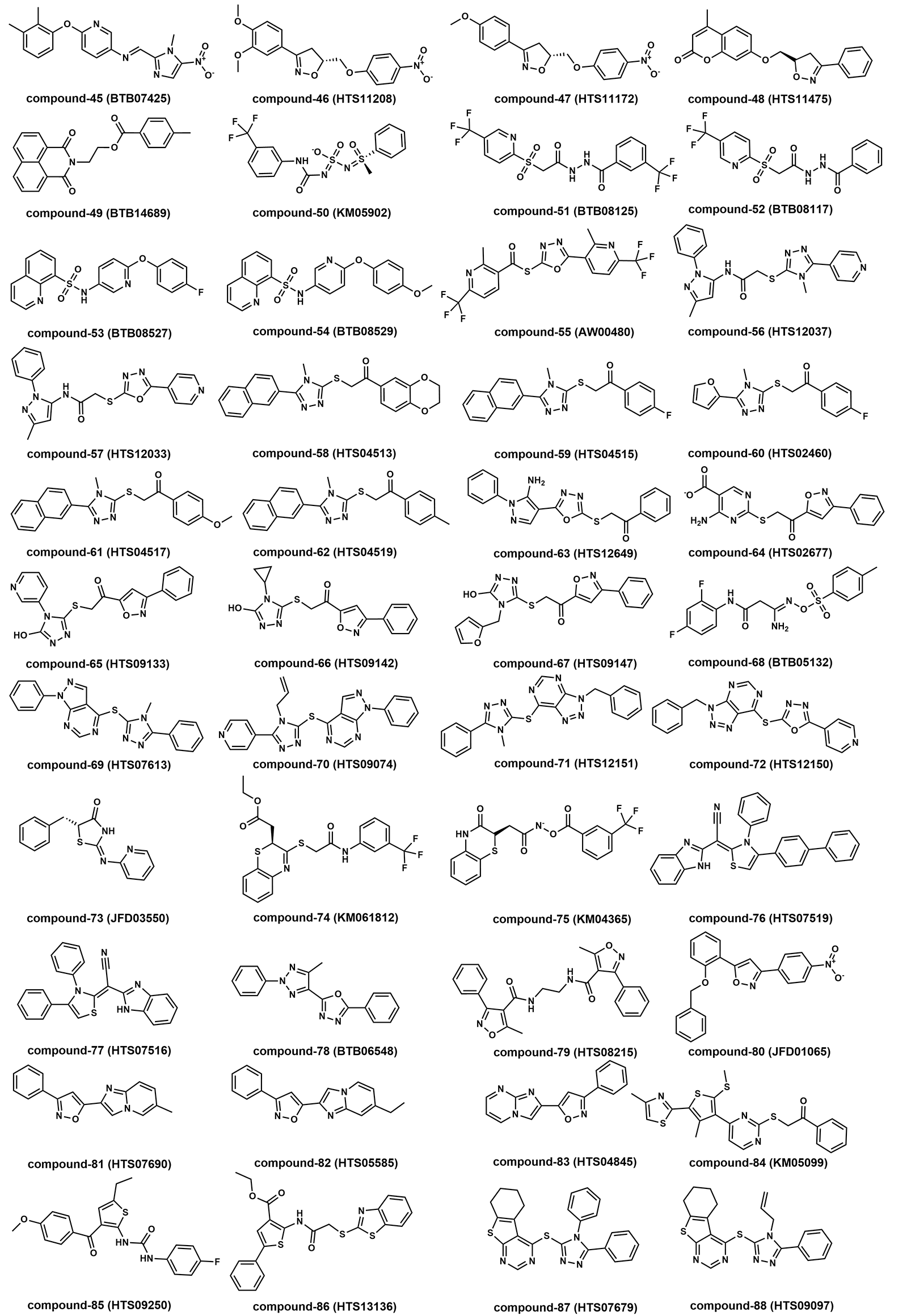


Figure S1.2. The structures of compounds 45 - 88 among the 116 compounds screened out by the pharmacophore model of MIF.


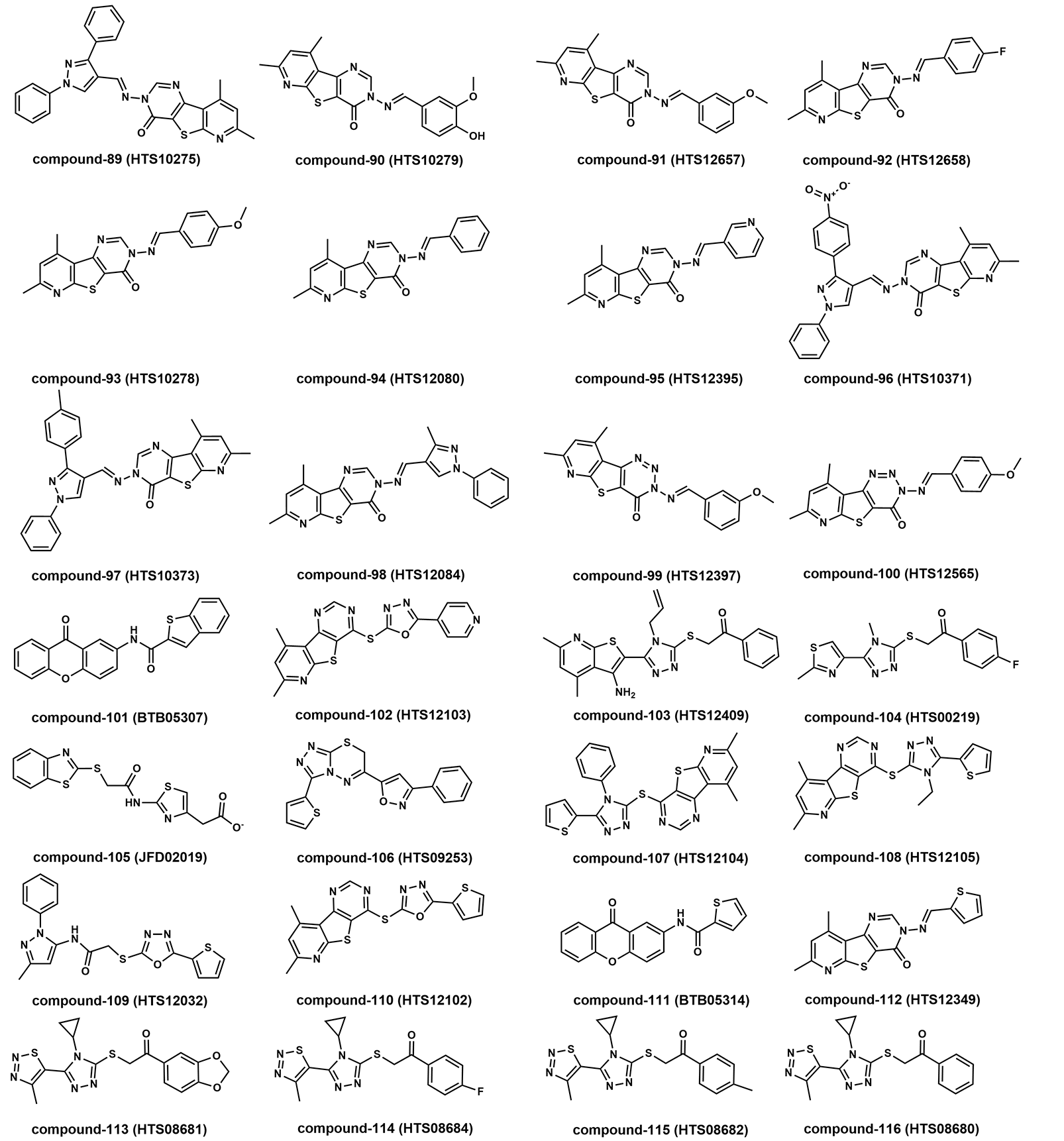


Figure S1.3. The structures of compounds 89 - 116 among the 116 compounds screened out by the pharmacophore model of MIF.

Table S1. The docking scores of the Hits 1-6 and compound 3a.

| **Name** | **Docking scores (kcal/mol)** |
| --- | --- |
| Hit-1 | -8.96 ± 0.61 |
| Hit-2 | -8.32 ± 0.58 |
| Hit-3 | -8.06 ± 0.52 |
| Hit-4 | -7.01 ± 0.55 |
| Hit-5 | -6.79 ± 0.48 |
| Hit-6 | -7.44 ± 0.41 |
| 3a | -6.32 ± 0.39 |


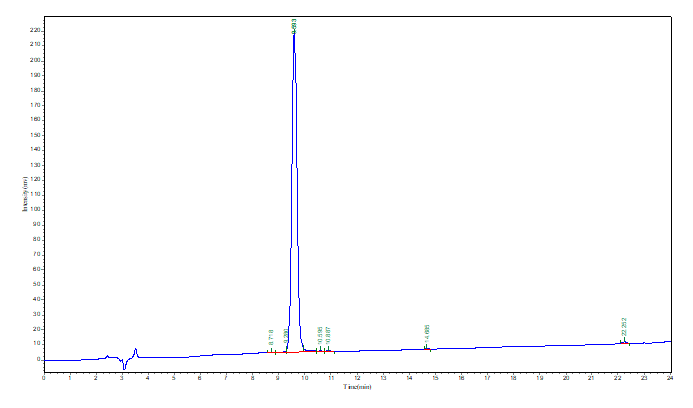


| Rank | Retention time (min) | Area | Conc |
| --- | --- | --- | --- |
| 1 | 8.718 | 3996.878 | 0.1326 |
| 2 | 9.280 | 7002.598 | 0.2322 |
| 3 | 9.593 | 2958116.750 | 98.1026 |
| 4 | 9.593 | 27745.717 | 0.9202 |
| 5 | 10.595 | 5737.362 | 0.1903 |
| 6 | 10.887 | 4369.561 | 0.1449 |
| 7 | 14.685 | 2148.002 | 0.0712 |
| 8 | 22.252 | 6211.405 | 0.2060 |
| Total |  |  | 100 |

Figure S2.1. The HPLC chromatogram of the Hit-1 (HTS05585).


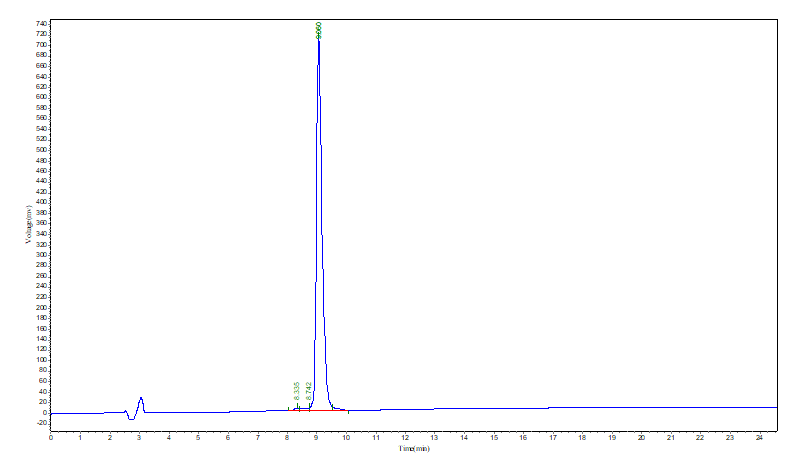


| Rank | Retention time (min) | Area | Conc |
| --- | --- | --- | --- |
| 1 | 8.335 | 53623.773 | 0.5583 |
| 2 | 8.742 | 66877.789 | 0.6963 |
| 3 | 9.060 | 9426473.000 | 98.1444 |
| 4 | 9.060 | 57727.465 | 0.6010 |
| Total |  |  | 100 |

Figure S2.2. The HPLC chromatogram of the Hit-2 (HTS07690).


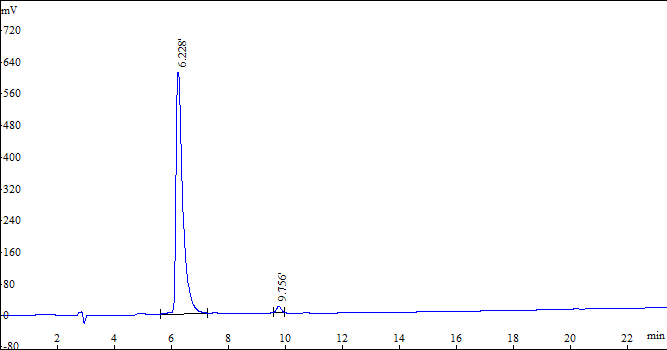


| Rank | Retention time (min) | Area | Conc |
| --- | --- | --- | --- |
| 1 | 6.228 | 9753889 | 98.4345 |
| 2 | 9.756 | 155128 | 1.5655 |
| Total |  |  | 100 |

Figure S2.3. The HPLC chromatogram of the Hit-3 (HTS04845).


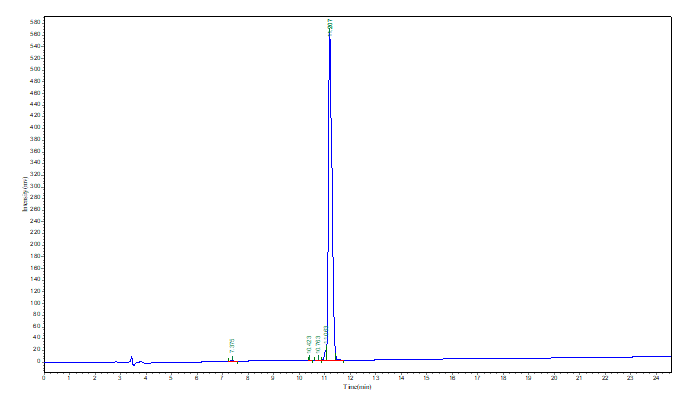


| Rank | Retention time (min) | Area | Conc |
| --- | --- | --- | --- |
| 1 | 7.375 | 6100.452 | 0.1125 |
| 2 | 10.423 | 2214.000 | 0.0408 |
| 3 | 10.763 | 2269.736 | 0.0419 |
| 4 | 11.063 | 69954.789 | 1.2899 |
| 5 | 11.207 | 5328772.500 | 98.2579 |
| 6 | 11.207 | 13940.919 | 0.2571 |
| Total |  |  | 100 |

Figure S2.4. The HPLC chromatogram of the Hit-4 (BTB06547).


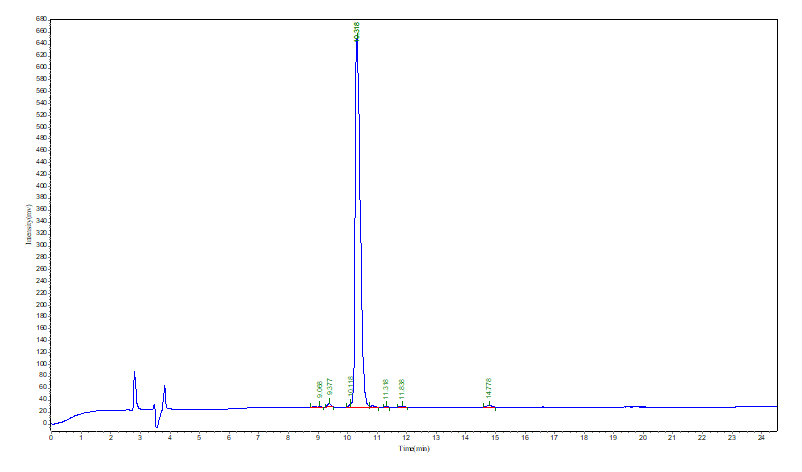


| Rank | Retention time (min) | Area | Conc |
| --- | --- | --- | --- |
| 1 | 9.068 | 9508.915 | 0.1274 |
| 2 | 9.377 | 41740.809 | 0.5591 |
| 3 | 10.118 | 20720.004 | 0.2775 |
| 4 | 10.318 | 7344209.500 | 98.3646 |
| 5 | 10.318 | 16630.434 | 0.2227 |
| 6 | 11.318 | 4860.795 | 0.0651 |
| 7 | 11.88 | 3641.606 | 0.0488 |
| 8 | 14.778 | 24999.965 | 0.3348 |
| Total |  |  | 100 |

Figure S2.5. The HPLC chromatogram of the Hit-5 (BTB06548).

| Rank | Retention time (min) | Area | Conc |
| --- | --- | --- | --- |
| 1 | 11.610 | 3729.900 | 0.0389 |
| 2 | 12.358 | 16938.398 | 0.1767 |
| 3 | 12.740 | 3707.687 | 0.0387 |
| 4 | 13.110 | 49317.578 | 0.5144 |
| 5 | 13.302 | 9412445.000 | 98.1727 |
| 6 | 13.302 | 39164.840 | 0.4085 |
| 7 | 14.288 | 62331.660 | 0.6501 |
| Total |  |  | 100 |

Figure S2.6. The HPLC chromatogram of the Hit-6 (HTS08258).

Table S2. Determination of the binding affinities of Hit-1 and 3a to MIF via an ITC assay.

| **Name** | **MIF (*K*_d_, µM)** |
| --- | --- |
| Hit-1 | 0.32 ± 0.01 |
| 3a | 0.94 ± 0.07 |


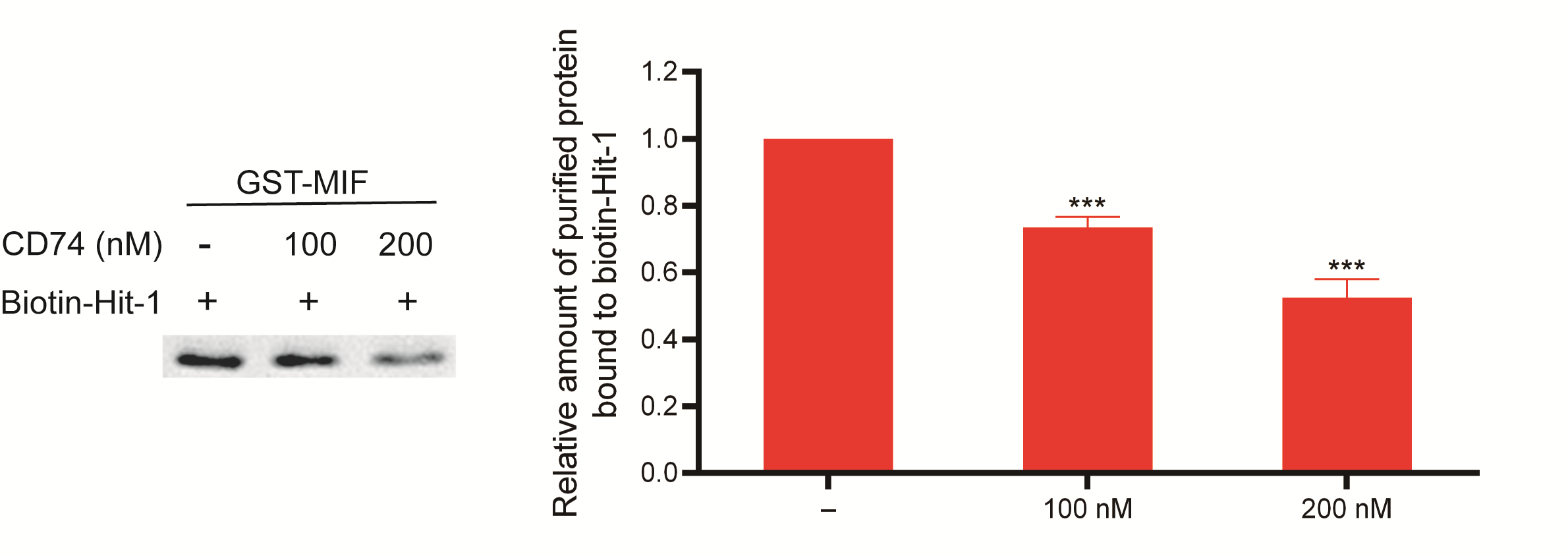


Figure S3. The relative amount of purified protein bound to biotin-Hit-1. Significance: ***p < 0.001
